# High Dose Local Photon Irradiation Is Crucial in Anti-CTLA-4 Antibody Therapy to Enhance the Abscopal Response in a Murine Pancreatic Carcinoma Model

**DOI:** 10.3390/cancers14092087

**Published:** 2022-04-22

**Authors:** Junya Yamamoto, Yutaka Takahashi, Kazumasa Minami, Keisuke Tamari, Shohei Katsuki, Wataru Takenaka, Shotaro Tatekawa, Kazuhiko Hayashi, Yuji Seo, Fumiaki Isohashi, Kazuhiko Ogawa, Masahiko Koizumi

**Affiliations:** 1 Department of Medical Physics and Engineering, Graduate School of Medicine, Osaka University, Suita, Osaka 565-0871, Japan; u414390j@gmail.com (J.Y.); k_minami@radonc.med.osaka-u.ac.jp (K.M.); skatsuki@sahs.med.osaka-u.ac.jp (S.K.); takewata216@gmail.com (W.T.); koizumi@sahs.med.osaka-u.ac.jp (M.K.); 2 Department of Radiation Oncology, Graduate School of Medicine, Osaka University, Suita, Osaka 565-0871, Japan; tamari@radonc.med.osaka-u.ac.jp (K.T.); s_tatekawa@radonc.med.osaka-u.ac.jp (S.T.); hayashi@radonc.med.osaka-u.ac.jp (K.H.); seo@radonc.med.osaka-u.ac.jp (Y.S.); isohashi@radonc.med.osaka-u.ac.jp (F.I.); kogawa@radonc.med.osaka-u.ac.jp (K.O.)

**Keywords:** radiation, immune checkpoint inhibitors, anti-CTLA-4 antibody, abscopal response, pancreatic ductal adenocarcinoma, regulatory T cell, cytotoxic T lymphocytes

## Abstract

**Simple Summary:**

Pancreatic cancer is an extremely treatment-resistant neoplasm to chemotherapy and immunotherapy. The combination of photon beam irradiation and anti-CTLA-4 antibody (C4) for the anti-tumor effect enhancement at local and distant tumors (abscopal tumors) was investigated using a pancreatic ductal adenocarcinoma (PDAC) mouse model. Monotherapy with C4 was not effective for PDAC. The high dose irradiation to local tumors produced significant shrinkage of irradiated tumors but did not induce the abscopal responses. In contrast, the combination therapy of high dose radiation and C4 enhanced the abscopal responses with significantly prolonged overall survival. The combination therapy dramatically decreased the regulatory T cell infiltration while increasing the cytotoxic T lymphocytes in the irradiated and abscopal tumors. These results suggest that high dose photon beam irradiation plays an important role in C4 therapy to enhance the abscopal response in PDAC.

**Abstract:**

Pancreatic cancer is an extremely treatment-resistant neoplasm to chemotherapy and immunotherapy. The combination of photon beam irradiation and anti-CTLA-4 antibody (C4) for the anti-tumor effect enhancement at local and distant tumors (abscopal tumors) was investigated using the pancreatic ductal adenocarcinoma (PDAC) mouse model. Pan02 cells were bilaterally inoculated to both legs of C57BL/6 mice. High dose photon beams in a hypofractionation or a single fraction were delivered to the tumors on one leg. Monotherapy with C4 via i.p. was not effective for PDAC. The high dose irradiation to the local tumors produced significant shrinkage of irradiated tumors but did not induce the abscopal responses. In contrast, the combination therapy of high dose photon beam irradiation in both hypofractionation and a single fraction with C4 enhanced the anti-tumor effect for abscopal tumors with significantly prolonged overall survival. The flow cytometric analysis revealed that the combination therapy dramatically decreased the regulatory T cell (Treg) proportion while increasing the cytotoxic T lymphocytes in both local and abscopal tumors. These results suggest that high dose photon beam irradiation plays an important role in C4 therapy to enhance the abscopal response with immune microenvironment changes in PDAC, regardless of the fractionation in radiation therapy.

## 1. Introduction

Most patients with pancreatic ductal adenocarcinoma (PDAC) are diagnosed at the advanced stage, and the 5-year survival rate of PDAC is only 8% [1]. The treatment of choice in metastatic PDAC is chemotherapy, but its treatment outcome is poor due to limited efficacy and life-threatening toxicities [1]. Additionally, metastatic pancreatic cancer is severely resistant to most treatments, including radiation therapy and chemotherapy [2].

Immune checkpoint molecules, such as cytotoxic T lymphocyte associated antigen-4 (CTLA-4) and programmed cell death-1 (PD-1) on the T cell surface, play a critical role in tumor immune escape [3,4]. Specifically, B7 and PD-ligand 1 (PD-L1) on the tumor surface bind to CTLA-4 and PD-1, respectively, thereby preventing the T cells from attacking the tumors [3,4]. Recently, immune checkpoint inhibitors (ICIs), such as an anti-CTLA-4 antibody (C4) and anti-PD-1 antibody, have dramatically improved the overall survival of patients even with stage IV cancers [5]. An anti-tumor response was obtained in 20–30% of patients with non-small cell lung cancer, renal cell carcinoma, and melanoma [5,6,7]; however, monotherapy with ICIs is ineffective for PDAC due to the intrinsic tumor microenvironments, in which cytotoxic T cells (CTL) are hindered to infiltrate into the tumors due to the thick stromal regions, and immune suppressive cells, such as regulatory T cells (Treg) and M2 macrophages, preferentially exist [8,9].

Recent preclinical and clinical data showed that local irradiation enhanced the anti-tumor effect for tumors not only inside the radiation field but also outside the radiation field, referred to as the abscopal effect [10,11,12]. One of the abscopal effect mechanisms is the radiation-induced danger signals, in which calreticulin (CRT) is translocated to the cell surface and high mobility group box-1 (HMGB-1) are released from dying cells, thereby facilitating dendritic cells to recognize tumor antigens, followed by T cell activation and clonal expansion [13,14,15]. An abscopal effect is a rare event in radiation therapy alone [12,16]; however, recent research demonstrated that the combination of ICIs with radiation therapy significantly increased the chances of the abscopal effect for several tumor types [12,17,18,19,20,21,22,23]. Additionally, no reports have investigated the effect of combination therapy with radiation and C4 on local and abscopal responses and the immune microenvironment changes in PDAC, which is known as an ICI refractory neoplasm.

Here, we show the enhanced immune response in combination therapy of high dose radiation both in a hypofraction and a single fraction with C4 using a PDAC mouse model. Furthermore, this study focused on the immune microenvironment changes that provide the rationale for ICI selection.

## 2. Materials and Methods

### 2.1. Cell Line

Pan02 mouse pancreatic adenocarcinoma cell line was purchased from the National Institutes of Health (Rockville, MD, USA). They were maintained in RPMI supplemented with 10% FBS and 5% penicillin/streptomycin and L-glutamine and incubated at 37 °C in a 5% CO₂ atmosphere in an incubator.

### 2.2. Ethics Statement

The mice were maintained in an area which is pathogen free at Osaka University, Suita, Japan. All experiments were approved by the Osaka University Institute Animal Care and Use Committee following the principles and procedures outlined in the Japanese Act on the Welfare and Management of Animals and Guidelines for the Proper Conduct of Animal Experiments issued by the Scientific Council of Japan (Approved #; 30-014-011). All interventions on animals were conducted to minimize pain. The mice were observed every 3 days and humanely sacrificed by CO_2_ when the longest diameter of the tumor was >20 mm, with the following criteria: prostration, skin lesions, and difficulty in breathing.

### 2.3. In Vitro Experiments

#### 2.3.1. Colony Formation Assay

Pan02 cells were plated on 6 cm dishes and maintained in an incubator overnight, and then irradiated at 0, 2, 4, 8, 10, 12, and 16 Gy with Gammacell 40 Exactor (Nordion, Ottawa, Canada). Ten days after culturing, these cells were washed with PBS, fixed with 10% formalin, and stained with crystal violet solution for 30 min. After washing three times, the colonies consisting of >50 cells were automatically counted by PSF-2000 (Shashin Kagaku Product Company, Kyoto, Japan). Surviving fraction was calculated based on the linear quadratic (LQ) model, which is expressed as “SF = exp (−αD −βD^2^)” [24], where SF, D, α, and β represent survival fraction, radiation dose, the incidence of double-strand breakage by one particle, and incidence of double-strand breakage by two particles, respectively.

#### 2.3.2. Enzyme-Linked Immunosorbent Assay (ELISA)

The cell culture supernatants were collected 72 h after irradiation. ELISA was performed using the Mouse/Rat HMGB1 ELISA kit (Arigo biolaboratories, Taiwan) to detect an amount of released HMGB-1 for 0 Gy (N = 3), 16 Gy (N = 4), and 8 Gy × 3 fr (N = 4).

To analyze cytoplasmic dsDNA, irradiated cells in the cytoplasm were isolated using NE-PERTM Nuclear and Cytoplasmic Extraction Reagents (Thermo Fisher Scientific, Waltham, MA, USA). Cytosolic dsDNA in 1 × 10^6^ of Pan02 cells was quantified using the ${\mathrm{SpectraMax}}^{®}$ ${\mathrm{Quant}}^{\mathrm{TM}}$ AccuClear Nano dsDNA Assay Explorer kit (Molecular Devices, San Jose, CA, USA) 24 h after the final irradiation.

We used the mouse interferon-beta (IFN-β) high-sensitivity ELISA kit (PBL Assay Science, Piscataway, NJ, USA) to quantify IFN-β in cell lysates collected 24 h after the final irradiation. The measured concentrations were normalized to the protein concentration by bicinchoninic acid assay (BCA) using the ${\mathrm{Pierce}}^{\mathrm{TM}}$ BCA protein assay kit (Thermo Fisher Scientific, USA). Samples were analyzed with Varioskan (Thermo Fisher Scientific, Waltham, MA, USA). All procedures were performed following the manufacturer’s manual.

#### 2.3.3. Western Blotting

Pan02 cells were lysed with ${\mathrm{Pierce}}^{\mathrm{TM}}$ RIPA buffer (Thermo Fisher Scientific, USA) with 1 × Protease/Phosphatase inhibitor (Thermo Fisher Scientific, USA) 24 h after the final irradiation. The supernatant was obtained by centrifugation at 12,000 rpm for 15 min at 4 °C. The protein concentration of each sample was measured by BCA assay.

Each sample mixed with sample buffer supplemented with 10% β-ME (Thermo Fisher Scientific) was placed on lanes of 10% unstained precast gel (Bio-Rad Laboratories, Hercules, San Diego, CA, USA) on SDS-PAGE. The electrophoresis was conducted at 75 kV for 10 min, followed by 100 kV for 1 h. Proteins were transferred to PVDF membranes for 50 min under 100 kV conditions. Blocking was performed using a blocking buffer (TBS-T containing 5% BSA or 5% skim milk) with shaking at room temperature for 1 h. The phosphorylated-IRF3 (p-IRF3) rabbit mAb (S396) (D601M, Danvers, MA, Cell Signaling Technologies) and cGAS rabbit mAb (Mouse Specific) (D3080, Cell Signaling Technologies, Danvers), diluted with TBS-T with 5% BSA or 5% skim milk at a ratio of 1:1250, were reacted at 4 °C overnight. HRP-linked anti-rabbit IgG antibody (Cell Signaling Technologies) diluted with blocking buffers at a ratio of 1:3000, was reacted for 1 h at room temperature. Super ${\mathrm{Signal}}^{\mathrm{TM}}$ West Pico PLUS Chemiluminescent Substrate (Thermo Fisher Scientific) was used for chemiluminescence, and bands were visualized utilizing the ChemiDoC Touch imaging systems (Bio-Rad, Hercules, CA, USA). Densitometric quantification of the Western blotting signals was executed using Image Lab (National Institutes of Health, Bethesda, MD, USA). The signals from each sample were normalized by the value of β-actin (Abcam, Cambridge, UK).

### 2.4. In Vivo Experiments

Six- to eight-week-old C57/BL/6 mice were purchased from Nihon-Clea (Tokyo, Japan). In the mixture of PBS and matrigel (1:1), 3 × 10⁵ Pan02 cells were inoculated to both legs of C57BL/6 mice. Every treatment was initiated when the longest diameter of the tumor exceeded 8 mm.

#### 2.4.1. Radiation Therapy

We analyzed the tumor volume changes in the following groups to investigate the anti-tumor effect of monotherapy with radiation: (1) no treatment group (NoTx; N = 12), (2) a group in which only one side leg of the tumor was irradiated at 8 Gy for 3 consecutive days from day 0 (8 Gy × 3 fr; N = 11), and (3) a group in which only one side leg of the larger tumor was irradiated with 16 Gy in a single fraction (16 Gy; N = 7). The irradiation was conducted using an orthovoltage X-ray irradiator under the condition of 180 kV, 15 mA, with a 1 mm Al filter or Gammacell 40 Exactor. The appropriate thickness of the lead shield blocks was placed to avoid unnecessary body exposures based on the film dosimetry. Tumor volumes of mice were calculated by the following formula “(Length) × (Width)^2^ × 0.52.” The length and the width were measured at least every 3 days.

#### 2.4.2. Combination Therapy of Radiation and C4

We analyzed the tumor volumes in the following groups to examine the effect of C4 with or without radiation: (1) a group without treatments (NoTx; N = 11), (2) a group treated with C4 (clone: 9H10, BioCell, Lebanon, NH, USA) on days 0, 3, and 6 (C4; N = 14), (3) a group treated with C4 and radiation 8 Gy × 3 fr from day 0 (8 Gy × 3 fr + C4; N = 8), and (4) a group treated with C4 and 16 Gy radiation (16 Gy + C4; N = 7). C4 at 150 μg was injected via i.p. on days 0, 3, and 6 for mice in C4, 8 Gy × 3 fr + C4, and 16 Gy + C4 groups.

The CD8 depletion experiment was performed in the following groups: (1) a group treated with C4 (N = 14), (2) a group treated with C4 with an anti-mouse CD8α antibody (C4 (CD8α); N = 8), (3) a group treated with 8 Gy × 3 fr + C4 with an anti-mouse CD8α antibody (8 Gy × 3 fr + C4 (CD8α); N = 8), and (4) a group treated with 16 Gy + C4 with an anti-mouse CD8α antibody (16 Gy + C4 (CD8α); N = 8). An anti-mouse CD8α antibody (InVivoMAb; clone: 2.43, BioCell, Lebanon, NH, USA) at 120 μg was administered via i.p. every 3 days starting at 9 days before tumor inoculation, based on a previous publication [18,20]. Treatment was initiated when the longest tumor diameter exceeded 8 mm (day 0). C4 was injected three times every 3 days.

#### 2.4.3. Flow Cytometry

The mice were sacrificed 9 days after the initial treatment to analyze the tumor immune microenvironments. Tumor samples were collected from the mice in the following groups: NoTx, C4, 8 Gy × 3 fr, 8 Gy × 3 fr + C4, 16 Gy, and 16 Gy + C4. For the CTL infiltration analysis in the irradiated tumors, the number of mice was 5, 5, 7, 4, and 5 in NoTx, C4, 8 Gy × 3 fr, 8 Gy × 3 fr + C4, 16 Gy, and 16 Gy + C4 groups, respectively. The corresponding number of mice in abscopal tumors was 5, 5, 4, 3, 4, and 4, respectively. For the Treg infiltration analysis in the irradiated tumors, the number of animals was 6, 6, 7, 4, 3, and 4 in NoTx, C4, 8 Gy × 3 fr, 8 Gy × 3 fr + C4, 16 Gy, and 16 Gy + C4 groups, respectively. The corresponding number of mice in the abscopal tumors was 6, 6, 4, 4, 4, and 4, respectively.

A single-cell suspension was prepared as previously described [20,22,25]. Briefly, tissue was dissociated with 1 mg/mL of Collagenase IV (Sigma Aldrich, Tokyo, Japan) and 0.2 mg/mL of DNase II (Sigma Aldrich) at 37 °C on a shaker for 20 min with pipetting every 10 min. After blocking Fc receptors with anti-CD16/32 antibody (clone: nr. 93, Biolegend, San Diego, CA, USA) for 10 min at room temperature in the dark, cells were incubated with anti-CD45-FITC antibody (clone: 30-F11, eBioscience, San Diego, CA, USA), anti-CD8-APC antibody (clone: 53-6.7, San Diego, eBioscience, CA, USA), anti-CD4-APC antibody (RM4-5, eBioscience, San Diego, CA, USA), anti-CD11b-APC antibody (clone: M1/70, eBioscience, San Diego, CA, USA), or anti-Ly-6G/Ly-6C-PE (clone: RB6-8C5, eBioscience, San Diego, CA, USA) on ice for 30 min at a 1:100 ratio with FACS buffer (PBS containing 10% FBS and 0.5 mM EDTA). For intracellular staining, cells were fixed, permeabilized using the FoxP3 staining kit (eBioscience), and reacted with anti-FoxP3-PE antibody (clone: FJK-16s, eBioscience, San Diego, CA, USA) or anti-Granzyme B-PE antibody (clone: NGZB, eBioscience, San Diego, CA, USA) on ice for 30 min at a 1:80 ratio. After washing twice with Perm buffer followed by washing once with FACS buffer, cells were analyzed with FACS verse flow cytometer (Beckton Dickinson, Franklin Lakes, NJ, USA). FlowJo version 10 software (Tommy Digital Biology, Tokyo, Japan) was used for data analysis.

### 2.5. Statistics

Tukey–Kramer’s HSD test was performed to compare the difference in cytosolic dsDNA between 0 Gy, 10 Gy, 16 Gy, and 8 Gy × 3 fr groups. Dunnett’s multiple comparison test was conducted for the comparison of the concentration of IFN-β between 0 Gy, 16 Gy, and 8 Gy × 3 fr groups. Tukey–Kramer’s HSD test was used to compare the differences in tumor volume and flow cytometry data between multiple groups. The overall survival was analyzed using the Kaplan–Meier method, and statistical significance was evaluated using the Wilcoxon test with Bonferroni correction. Tumor volume was evaluated until 70% of the mice in each group were dead. *p*-values of <0.05 were considered significantly different.

## 3. Results

### 3.1. Radiation Sensitivity of PDAC

We previously demonstrated that the anti-PD-L1 antibody and C4 with photon irradiation at 10 Gy, which obtains 0.5% survival of mouse osteosarcoma cell line (LM8), produced the enhanced anti-tumor effect in local and abscopal sites [21,22]. Similarly, we calculated the γ-ray dose to obtain a 0.5% survival fraction in the Pan02 cell line based on the LQ model [24].

**Figure 1:**
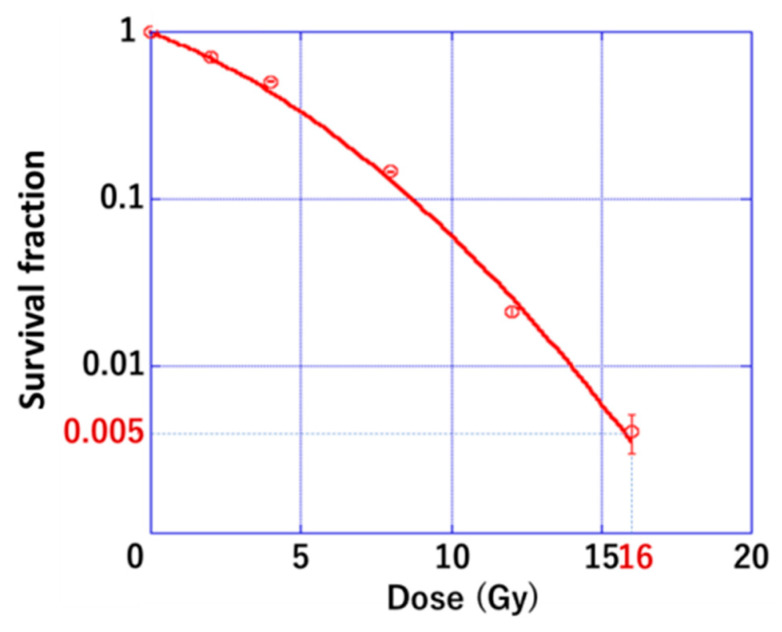
A cell survival curve of Pan02 cell line.

As shown in Figure 1, 16 Gy in a single fraction provided 0.5% survival. Therefore, 16 Gy was used in a single fraction, or its biological equivalent, 8 Gy × 3 fr, which was estimated by the concept of biological effective dose with the LQ model [26]. The detail of the derivation of the hypofractionated radiation dose from 16 Gy in a single fraction is provided in the Supplementary Materials.

### 3.2. Danger Signal Was Induced in a Dose-Dependent Manner

Tumor irradiation induces the danger signal, including a CRT translocation to the cell surface, and releases the damage-associated molecule patterns (DAMPs), such as HMGB-1, which promotes tumor antigen recognition by dendritic cells and T cell activation [13,14,15,27]. Several groups demonstrated that the danger signal induction is associated with the abscopal effect [14,16,27]. We examined the optimal radiation delivery regimen (e.g., total dose, fractionation) to effectively induce the danger signal for PDAC in vitro.

**Figure 2:**
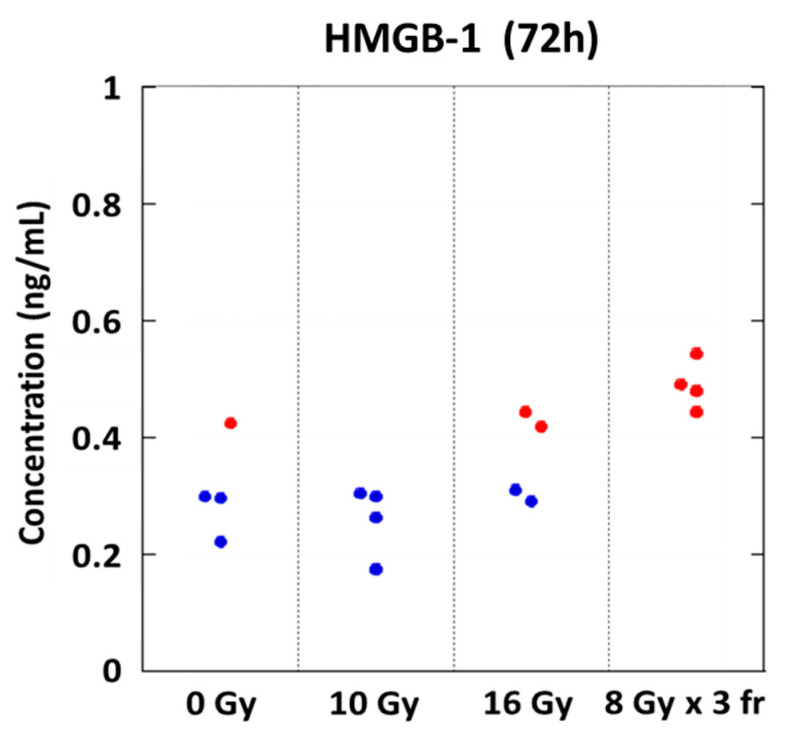
Release of HMGB-1 at 0 Gy (N = 4), 10 Gy (N = 4), 16 Gy (N = 4), and 8 Gy × 3 fr (N = 4). Blue circles represent data below the detection level in the ELISA kit. Abbreviations—HMGB-1: high mobility group box-1; fr: fraction.

The release of HMGB-1 was increased in a dose-dependent manner. The highest release was observed at 8 Gy × 3 fr 72 h after irradiation (Figure 2).

### 3.3. Radiation Dose Escalation Induces the Type-I Interferon Pathway Activation

**Figure 3:**
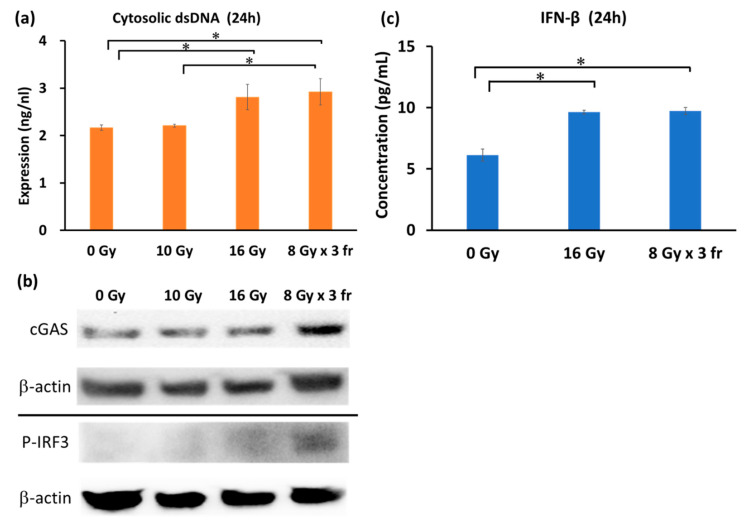
Analysis of the type-I interferon pathway after irradiation. (**a**) The cytosolic dsDNA concentration 24 h after the final irradiation. Tukey–Kramer’s HSD test was performed to compare the difference between 0 Gy (N = 7), 10 Gy (N = 7), 16 Gy (N = 8), and 8 Gy × 3 fr (N = 8). (**b**) The expression levels of proteins in the type-I interferon pathway 24 h after the final irradiation. Untrimmed data are provided in Supplementary Figure S1. (**c**) The IFN-β concentration in cell lysates 24 h after the final irradiation at 0 Gy (N = 3), 16 Gy (N = 4), and 8 Gy × 3 fr (N = 4). Dunnett’s test was performed to compare the difference in the expression level of IFN-β between 0 Gy vs. 16 Gy and 0 Gy vs. 8 Gy × 3 fr. Each error bar represents SE. *: *p* < 0.05. Abbreviation—fr: fraction.

The type-I IFN pathway leads to tumor-specific-antigen presentation promotion [28,29,30,31,32]. A previous report demonstrated high dose radiation in a hypofractionation but not in a single fraction activated type-I IFN pathway, and increased the secretion of IFN-β breast cancer and colon cancer cell lines [17,22]. Therefore, we investigated the dose and fractionation dependences on the expression changes of proteins related to the type-I IFN pathway in vitro. The irradiation at 16 Gy in a single fraction and 8 Gy × 3 fr significantly increased the cytosolic double-stranded DNA (dsDNA) level compared with 0 Gy (Figure 3a).

The expression level of cGAS showed the maximum expression level at 8 Gy × 3 fr. The average area density values normalized to a loading control (β-actin) in three independent experiments were 0.527 ± 0.168, 0.716 ± 0.207, 0.670 ± 0.181, and 1.283 ± 0.553, at 0 Gy, 10 Gy, 16 Gy, and 8 Gy × 3 fr, respectively. The expression level of phosphorylated-IRF3 (p-IRF3), which plays an important role in the intracellular signal transduction cascade, increased in a dose-dependent manner, but only a slight difference was observed between the IFN-β concentration in the cell lysates 24 h after the last irradiation increased at 16 Gy and 8 Gy × 3 fr compared with 0 Gy (Figure 3c).

### 3.4. Radiation Strongly Inhibited PDAC Growth in Irradiated Tumors but Not in Abscopal Tumors

**Figure 4:**
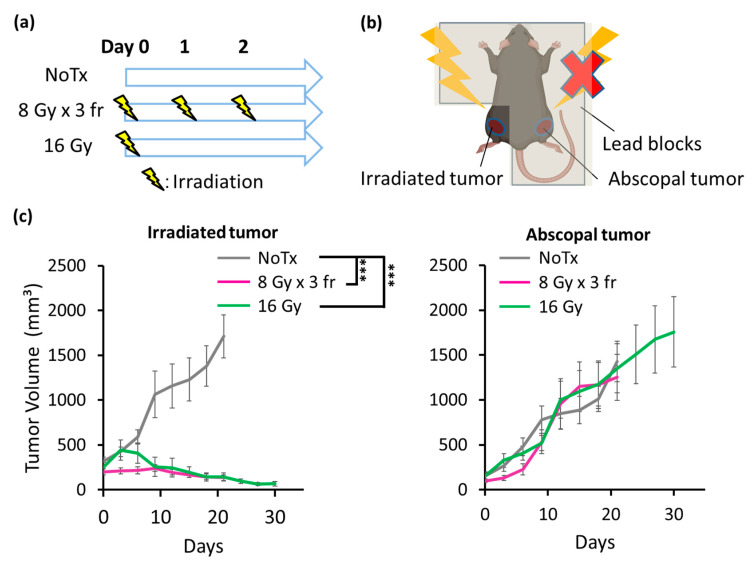
Tumor volume changes by radiation monotherapy. (**a**) The treatment schedule. Treatments were initiated when the longest diameter of the tumor exceeded 8 mm (day 0). (**b**) The irradiation setup. (**c**) The tumor volume changes in irradiated and abscopal tumors. Each error bar represents SE. Tukey–Kramer’s HSD test was performed to compare the differences in tumor volumes on day 18 between NoTx (N = 12), 8 Gy × 3 fr (N = 11), and 16 Gy (N = 7) groups. Tumor volume was evaluated until 70% of the mice in each group were dead. ***: *p* < 0.001. Abbreviations—NoTx: no treatment; fr: fraction.

The danger signal and type-I IFN pathway were induced by high dose photon irradiation both in a hypofractionation and in a single fraction; therefore, we next examined whether radiation inhibits tumor growth at the local and abscopal sites using the bilateral tumor-bearing mouse model (Figure 4a,b). Photon beams were irradiated to the tumors on one leg while the rest of the body was shielded by enough thickness of lead blocks when the longest diameter of the tumors reached ≥8 mm.

Significant tumor growth inhibition was observed in the irradiated tumors by 16 Gy and 8 Gy × 3 fr compared with the NoTx group. However, no significant reduction was found in the abscopal tumor volume by monotherapy with high dose irradiation (Figure 4c).

### 3.5. Combination Therapy of Radiation and C4 Inhibited Tumor Growth Both in Irradiated and Abscopal Sites

**Figure 5:**
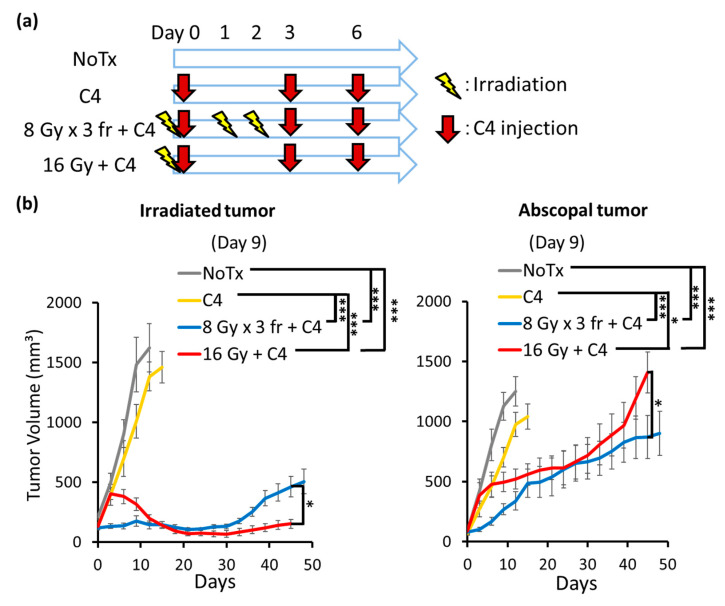
Tumor volume changes. (**a**) Treatment schedules. Treatments were initiated when the longest tumor diameter exceeded 8 mm (day 0). (**b**) The tumor volume changes in irradiated and abscopal tumors. Each error bar represents SE. Tukey–Kramer’s HSD test was performed to compare the difference in the tumor volume between the NoTx (N = 11), C4 (N = 14), 8 Gy × 3 fr + C4 (N = 8), and 16 Gy + C4 (N = 7) groups on day 12. The Student’s *t*-test was performed to compare the differences in the tumor volume between 8 Gy × 3 fr + C4 (N = 7) and 16 Gy + C4 (N = 4) on day 45. Tumor volume was evaluated until 70% of the mice in each group were dead. ***: *p* < 0.001, *: *p* < 0.05. Abbreviations—NoTx: no treatment; C4: anti-CTLA-4 antibody; fr: fraction.

The tumor growth delay was not observed in the abscopal tumors by monotherapy with radiation, thus we next examined whether the addition of C4 to radiation induces a stronger anti-tumor effect. Photon beam irradiation and C4 injection were simultaneously initiated when the longest diameter of tumors reached ≥8 mm (Figure 5a).

C4 monotherapy was ineffective for PDAC. Local irradiation suppressed tumor growth in the irradiated tumors compared with C4. The local effect was observed both in a single fraction and hypofractionation (Figure 5b), which was similar to monotherapy with radiation therapy (Figure 4c). A comparison of the irradiated tumor volume revealed that significant tumor growth delay was observed both at 16 Gy + C4 and 8 Gy × 3 fr + C4 groups by day 30. However, the significant regrowth of the irradiated tumor was observed in the mice in the 8 Gy × 3 fr + C4 group.

Interestingly, the significant abscopal tumor volume reduction was obtained by the combination therapy of both radiation delivery regimens. Of note, the local and abscopal responses in the 8 Gy × 3 fr + C4 group were initiated earlier than that of the 16 Gy + C4 group. A similar abscopal response was observed until day 40 in both groups; however, rapid tumor regrowth was observed in the 16 Gy + C4 group (*p* < 0.05) (Figure 5b).

### 3.6. Local Irradiation Altered Immune Microenvironments Both in Irradiated and Abscopal Tumors

**Figure 6:**
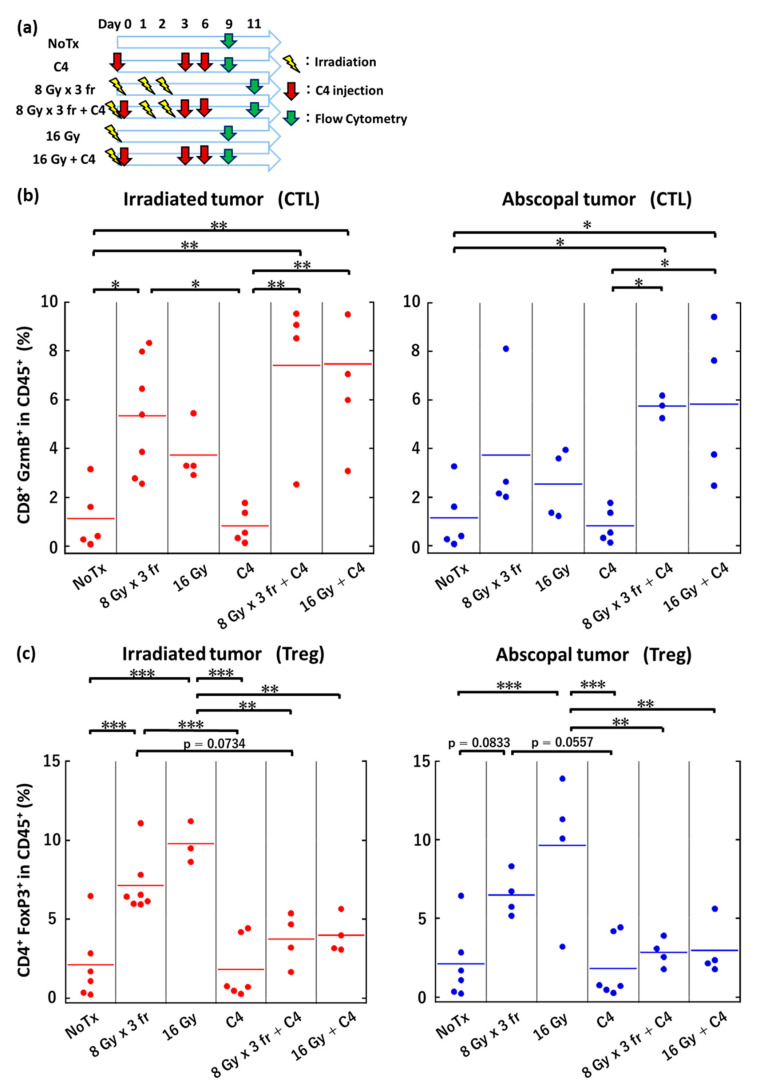
The immune microenvironment changes in tumors by combination therapy of radiation and C4. (**a**) The treatment schedule. (**b**) Dot plots showing the proportion of CTL on day 9 in the irradiated tumors in NoTx (N = 5), C4 (N = 5), 8 Gy × 3 fr (N = 7), 8 Gy × 3 fr + C4 (N = 4), 16 Gy (N = 4), and 16 Gy + C4 (N = 5) groups and in the abscopal tumors in NoTx (N = 5), C4 (N = 5), 8 Gy × 3 fr (N = 4), 8 Gy × 3 fr + C4 (N = 3), 16 Gy (N = 4), and 16 Gy + C4 (N = 4) groups. (**c**) Dot plots showing the proportion of Treg on day 9 in the irradiated tumors in NoTx (N = 6), C4 (N = 6), 8 Gy × 3 fr (N = 7), 8 Gy × 3 fr + C4 (N = 4), 16 Gy (N = 3), and 16 Gy + C4 (N = 4) groups and in the abscopal tumors in NoTx (N = 6), C4 (N = 6), 8 Gy × 3 fr (N = 4), 8 Gy × 3 fr + C4 (N = 4), 16 Gy (N = 4), and 16 Gy + C4 (N = 4) groups. Representative flow cytometry dot plots of CTL and Tregs are provided in Figure S2a,b, respectively. Tukey–Kramer’s HSD test was used to compare CTL and Treg between multiple groups. ***: *p* < 0.001, **: *p* < 0.01, *: *p* < 0.05. NoTx: no treatment; C4: anti-CTLA-4 antibody; CTL: cytotoxic T lymphocytes; Treg: regulatory T cell; fr: fraction.

A significant anti-tumor effect was observed in the irradiated and abscopal tumors, thus we next examined the immune microenvironment including CTL (CD8⁺, Granzyme B⁺ (GzmB⁺) in CD45⁺) and Treg (CD4⁺, FoxP3⁺ in CD45⁺). The flow cytometric analysis revealed that local irradiation at 8 Gy *×* 3 fr significantly increased the CTL recruitment in the irradiated tumors, whereas a moderate increase was obtained at 16 Gy in a single fraction (Figure 6b). Interestingly, the same trend was observed in the abscopal tumors (Figure 6b).

However, the infiltration level of Tregs was also significantly increased after irradiation at 8 Gy *×* 3 fr or 16 Gy in a single fraction of both irradiated and abscopal tumors (Figure 6c).

The myeloid derived suppressor cell (MDSC) infiltration was not changed by local irradiation (Figure S3).

C4 is known to deplete Tregs, thus we further investigated the immune microenvironment changes by the combination therapy of radiation and C4. C4 monotherapy failed to recruit CTL to tumors. In contrast, the combination therapy significantly increased the CTL infiltration (Figure 6b) both in irradiated and abscopal tumors.

Radiation increased Treg accumulation in irradiated tumors both at 16 Gy in a single fraction and 8 Gy *×* 3 fr. These increases were abolished by the combination of C4. A similar trend was observed in abscopal tumors. A higher radiation-induced Treg was found in 16 Gy in a single fraction, while the moderate Treg increase was observed in 8 Gy *×* 3 fractions. This radiation-induced Treg was reduced by the combination therapy (Figure 6c).

### 3.7. The Anti-Tumor Effect of Combination Therapy of Radiation and C4 Was Attributed to CD8⁺ T Cells

**Figure 7:**
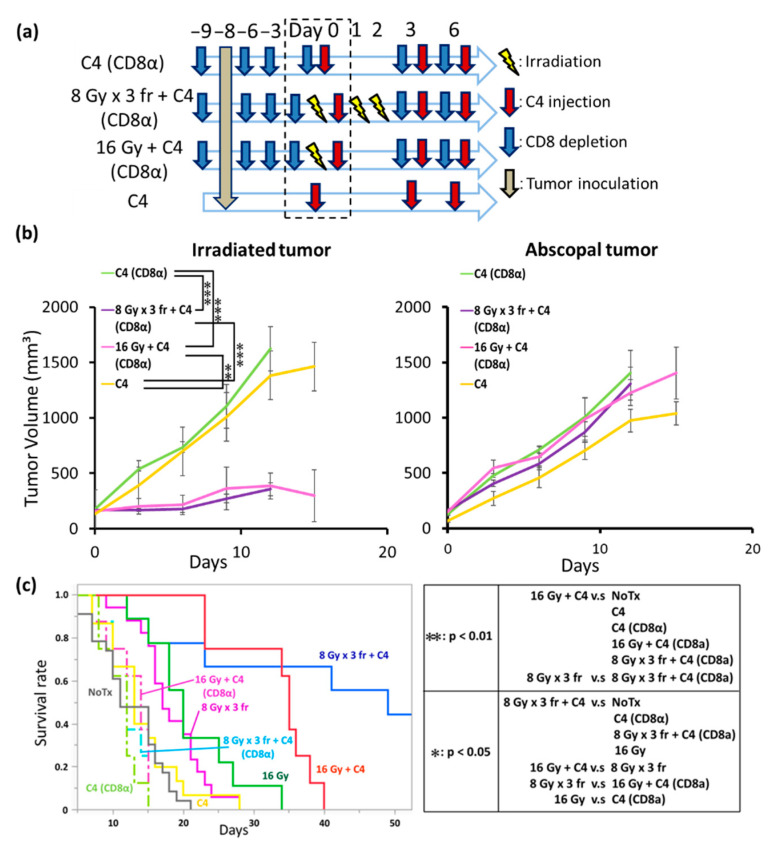
CD8 depletion experiments. (**a**) The experimental scheme. (**b**) The tumor volume changes of the irradiated and abscopal tumors. Tukey–Kramer’s HSD test was performed 15 days after the final treatment to compare the differences in tumor volume between C4 (CD8α) (N = 8), 8 Gy × 3 fr + C4 (CD8α) (N = 8), 16 Gy + C4 (CD8α) (N = 8), and C4 (N = 14) groups. The data of the C4 group are shared in Figure 5. (**c**) Overall survival. The Wilcoxon test with a post adjustment test by Bonferroni correction was performed to compare the difference among 9 groups. (NoTx, N = 23; C4, N = 14; 8 Gy × 3 fr, N = 11; 8 Gy × 3 fr + C4, N = 8; 16 Gy, N = 7; 16 Gy + C4, N = 7; C4 (CD8α), N = 8; 8 Gy × 3 fr + C4 (CD8α), N = 8; 16 Gy + C4 (CD8α), N = 8). The data of NoTx, C4, 8 Gy × 3 fr, 16 Gy, 8 Gy × 3 fr + C4, and 16 Gy + C4 groups are shared in Figure 4 and Figure 5. Each error bar represents SE. ***: *p* < 0.001, **: *p* < 0.01, *: *p* < 0.05. Abbreviations—C4: anti-CTLA-4 antibody; CD8α: anti-CD8 antibody; C4 (CD8α): anti-CTLA-4 antibody with the anti-CD8 antibody; 8 Gy × 3 fr + C4 (CD8α): 8 Gy × 3 fraction with anti-CTLA-4 and anti-CD8 antibodies; 16 Gy + C4 (CD8α): with anti-CTLA-4 and anti-CD8 antibodies; fr: fraction.

We administered a CD8 depletion antibody (CD8α), as shown in Figure 7a, to determine whether CD8⁺ T cells contribute to the anti-tumor effect in local and abscopal sites in the combination therapy. CD8 depletion antibody was administered every 3 days from the day before tumor inoculation (day 9), and thereafter every 3 days by 6 days after the start of treatment (day 6) based on a previous publication [18,20]. Irradiated tumor growth was still reduced in the combination of high dose irradiation with C4 even under CD8α administration. In contrast, CD8α administration completely abolished the enhanced abscopal responses (Figure 7b). The median survival period in the NoTx and C4 groups were almost identical, which were 11 and 13 days, respectively. In contrast, the addition of C4 to 16 Gy or 8 Gy × 3 fr significantly prolonged the overall survival with a median survival of 35 and 49 days, respectively, although the difference was not statistically significant. These survival benefits were abolished by CD8α (Figure 7c).

## 4. Discussion

The standard treatment for PDAC includes surgery, chemotherapy, radiation therapy, and their combination. However, their treatment outcomes have poor overall survival, especially at the advanced stage. ICIs have attracted attention for a variety of advanced tumors [5,6,7]; however, the response rate for patients with PDAC has remained low due to the unique tumor microenvironments, including thick stroma, and the abundance of immune suppressive cells [8,9]. However, the effect of C4 in combination with local radiation on anti-tumor efficacy at irradiated and abscopal tumors was unclear for PDAC. To our best knowledge, this is the first report that demonstrated the combination therapy of high dose local irradiation with C4, which revealed the local and systemic anti-tumor effect and alters the tumor immune microenvironments not only in irradiated but also in the abscopal tumors in a PDAC mouse model.

First, we found that PDAC cell line irradiation induced the danger signal, such as CRT and HMGB-1, in a dose-dependent manner. Previous studies demonstrated that tumor cell irradiation induces CRT translocation to the cell surface and the release of HMGB-1 in vitro, in vivo, and in humans [12,13,14,15,16,19,33], which promotes phagocytosis of dying tumor cells by dendritic cells through toll-like receptor 4 and mediates the cross-presentation of tumor antigens to CD8 T cells [13,14,15]. Most in vitro and in vivo studies evaluated the danger signal induction by radiation in a single fraction, thus we evaluated the effect of fractionation of a radiation delivery that effectively induces the danger signal. Our data revealed that hypofractionated irradiation releases higher HMGB-1 than single irradiation.

The type-I IFN pathway activation is another factor that enhances the radiation-induced immune response [17,22,32,34]. The tumor irradiation induces DNA damage, resulting in cGAS-STING pathway activation through sensing of cytosolic dsDNA fragments translocated to the cytoplasm [17,22,32,34]. Vanpouille-Box et al. demonstrated that the accumulation of dsDNA, the expression level of proteins related to the cGAS-STING pathway, and the release of IFN-β were induced by high dose hypofractionated irradiation but not by a high radiation dose in a single fraction in murine breast cancer and colorectal carcinoma models [17]. Similarly, our results revealed that the expression of cGAS by hypofractionated irradiation was greater than those by a single fraction. However, unlike the previous report [17], the expression change of p-IRF3 by hypofractionated irradiation was similar to the biologically equivalent dose in a single fraction. Accordingly, the elevated IFN-β expression was found both at hypofractionated and single fractionated radiation deliveries, compared with 0 Gy. To support this, our group recently demonstrated that high dose irradiation is necessary to promote the type-I IFN pathway and induce the abscopal response, which was independent of radiation delivery fractionation in a murine osteosarcoma model [22]. Taken together, our data suggest that high dose local irradiation is necessary to induce a stronger immune response, regardless of fractionation in radiation delivery in the PDAC model.

Second, we found that the combination therapy of radiation and C4 enhanced the anti-tumor effect for the abscopal tumors and prolonged the overall survival, which was independent of fractionation in radiation delivery. The recent innovation of radiation therapy has improved the local control of various tumor types. However, the role of radiation for PDAC is controversial due to its resistance to radiotherapy [35,36]. Seifert et al. reported that high dose irradiation of over 6 Gy to PDAC promoted tumor growth due to the promotion of fibrosis, which significantly hinders T cell infiltration and facilitates the requirement of immunosuppressive Treg and M2 macrophages [35]. Monotherapy with ICIs is also not effective for PDAC because of thick stroma and treatment-induced fibrosis [8]. Therefore, we hypothesized that the combination of radiation and ICIs provides at least an additive effect for local control and increased abscopal response of PDAC. Our results confirmed that C4 monotherapy did not provide therapeutic gains. A previous study demonstrated that irradiation at 10 Gy combined with PD-L1 mAb did not inhibit tumor growth due to the stimulation of colony-stimulating factor [36], thus our study utilized C4 and high dose local irradiation, which successfully produced both local and abscopal responses.

The DAMPs and type-I IFN pathway were strongly induced by radiation both in a hypofractionated and a single fraction delivery in the present study, thus we sought the optimal radiation delivery regimen in combination with C4 to induce the immune response. A previous report demonstrated that tumor irradiation at an extremely high dose (e.g., 20 Gy) induced the higher amount of HMGB-1 release [37]; up to 16 Gy or its biologically equivalent dose, 8 Gy × 3 fr, was used to avoid normal tissue toxicity in vivo. Our results demonstrated that the high dose radiation monotherapy was adequate to inhibit irradiated tumor progressions until day 30, although further follow-up beyond day 30 was not conducted due to the rapid growth in unirradiated tumors (Figure 4c). The same level of irradiated tumor volume inhibition was observed in C4 in combination with high dose radiation, indicating that the additive effect of C4 was masked by the adequate tumor regression by direct radiation damage to tumors. Importantly, the hypofractionated radiation at 8 Gy × 3 fr + C4 induced a local response earlier than 16 Gy in a single fraction + C4; the therapeutic gain was similar between 8 Gy × 3 fr + C4 and 16 Gy + C4 for a while (e.g., until day 30), and then only 16 Gy + C4 maintained irradiated tumor growth inhibitions for a long term. In contrast, 8 Gy × 3 fr + C4 failed to prevent local recurrence (Figure 5b).

Interestingly, long-term follow-up revealed that a stronger abscopal response was obtained by 8 Gy × 3 fr + C4 than by 16 Gy + C4, which was opposite to the local response. This likely led to that there was no statistical difference in the overall survival between 8 Gy × 3 fr + C4 and 16 Gy + C4 groups (Figure 7c). However, both groups achieved prolonged overall survival compared with C4 monotherapy. Taken together, our data suggest that high dose irradiation is required in combination with C4 for PDAC, but it did not depend on radiation therapy fractionation. To support this, another group reported that the combination of irradiation at 12 Gy in a single fraction with an anti-PD-L1 antibody enhanced the local effect [38].

Because of the nature of in vivo experiments, large uncertainty exists. Thus, we had mice in the NoTx and C4 groups in all separate tumor volume experiments (Figure 4 and Figure 5). For the radiation monotherapy and the combination therapy groups, radiation was delivered to legs with larger tumors. Thus, to make a fair comparison in tumor volumes, we compared the larger tumor in the NoTx and C4 groups with the irradiated tumor in the radiation monotherapy and combination therapy groups, and the smaller tumor in the NoTx and C4 groups with the abscopal tumor in the radiation monotherapy and combination therapy groups.

Finally, we found that tumor irradiation increased the infiltration of Treg and CTL in PDAC. In the present study, the number of mice analyzed in flow cytometry ranged from three to seven, depending on the treatment group. This was because of the inadequate number of cells obtained due to the treatment response of irradiated tumors by monotherapy with radiation and both irradiated and unirradiated tumors by the combination therapy, poor tumor engraftment, and sample preparation issues. Nevertheless, the trend, in which both CTL and Treg were increased by radiation and CTL was increased with Treg decrease, is clear with statistical significance.

The positive prognostic role in high CD8⁺ T cell infiltration after high dose irradiation has been consistently demonstrated in several malignancies [38,39]. However, the irradiation of PDAC has a significant impact on the CTL due to the increasing proportion of immunosuppressive Treg and M2 macrophages, leading to tumor progression [35,40]. Similarly, we found that monotherapy with radiation increased Treg infiltration despite the increased CTL infiltration. CTL was hindered to reach the tumor cells, which was different from melanoma because of the abundance of the thick stroma in PDAC, as well as the preferential existence of Treg and M2 macrophages [8,9]. However, our results imply that high dose radiation may contribute to tumor microenvironmental changes, including stroma cells, although its underlying mechanisms remained unclear. Additionally, our data suggest that radiation plays a role not only in immune enhancement but also in immune evasion (Treg accumulation) for PDAC. Under this condition, C4 can be rationalized as the first therapeutic option in combination with radiation because C4 suppresses Treg functions and CD8-mediated immune enhancement [41]. To support this hypothesis, our data revealed that the addition of C4 to radiation abolished radiation-induced Treg accumulations, while CTL was kept at the same level both in irradiated and abscopal tumors. Importantly, regrowth in irradiated tumors and rapid regrowth in the abscopal tumors occurred at similar timing (e.g., on day 39). To confirm the enhanced local and abscopal responses are a CD8-mediated phenomenon, we administrated a CD8 depletion antibody for mice which received combination therapy of high dose radiation and C4. We found that CD8⁺ T cell depletion abolished the abscopal response and the survival benefit of the combination of radiation and C4.

The pancreas is surrounded by highly radiosensitive organs, such as the digestive tract. Under this situation, fractionated irradiation is favorable to reduce the adverse events to normal tissues. Thus, the combination therapy of fractionated irradiation and C4 is superior for PDAC.

This experiment was conducted in a subcutaneous inoculation model, in which Pan02 cells were inoculated under the skin of the legs. The subcutaneous inoculation model reflects, to some extent, the tumor microenvironment unique to pancreatic cancer cells, such as a large number of stroma and the occupancy of suppressive immune cells; thus, the clinical situation is not fully reproduced. To solve this issue, we are currently working on a project using a PDAC orthotopic transplantation model, which will bring our findings closer to clinical practice.

## 5. Conclusions

High dose photon beam irradiation plays an important role in C4 therapy to enhance the abscopal response with immune microenvironment changes in PDAC, regardless of radiation therapy fractionation. There is no effective therapeutic option for PDAC, especially in the advanced stage. Our data provide a rationale for further studies to bring this combination therapy to clinical trial.

## Data Availability

The data presented in this study are available within the article and its Supplementary information files or upon reasonable requests to the corresponding author.

## Supplementary Materials

The following supporting information can be downloaded at: https://www.mdpi.com/article/10.3390/cancers14092087/s1, Supplemental Methods S1. Derivation of the biological equivalent dose of 16 Gy in a single fraction. Figure S1. Untrimmed data of Western blotting in Figure 3b. Figure S2. Representative dot plots in flow cytometry analyzing (a) cytotoxic T cells (CD8⁺-APC and GzmB⁺-PE) and (b) regulatory T cells (CD4+-APC and FoxP3-PE). Figure S3. The proportion of MDSC in each group. (a) Schedule of the experiment. Treatments were initiated when the longest diameter of the tumor exceeded 8 mm (day 0). (b) The proportion of MDSC in each group in irradiated and abscopal tumors. Error bars represent SE. The Tukey–Kramer HSD test was performed to compare the differences in the proportion of MDSC between the NoTx, 8 Gy × 3 fr, and 16 Gy. (c) Representative dot plots of MDSC analysis. MDSC is defined as CD11b^high^ Gr-1⁺ cells. Abbreviations—C4: anti-CTLA-4 antibody, MDSC: myeloid derived suppressor cells, fr: fraction.
