# Supplementary material for: High Dose Local Photon Irradiation Is Crucial in Anti-CTLA-4 Antibody Therapy to Enhance the Abscopal Response in a Murine Pancreatic Carcinoma Model
Source: Cancers (Basel). 2022 Apr 22;14(9):2087. doi: 10.3390/cancers14092087 (PMC9101709; doi:10.3390/cancers14092087)
Supplement: Supplementary file 1 [file cancers-14-02087-s001.zip › cancers-1660363-supplementary Figures.pdf]

cancers

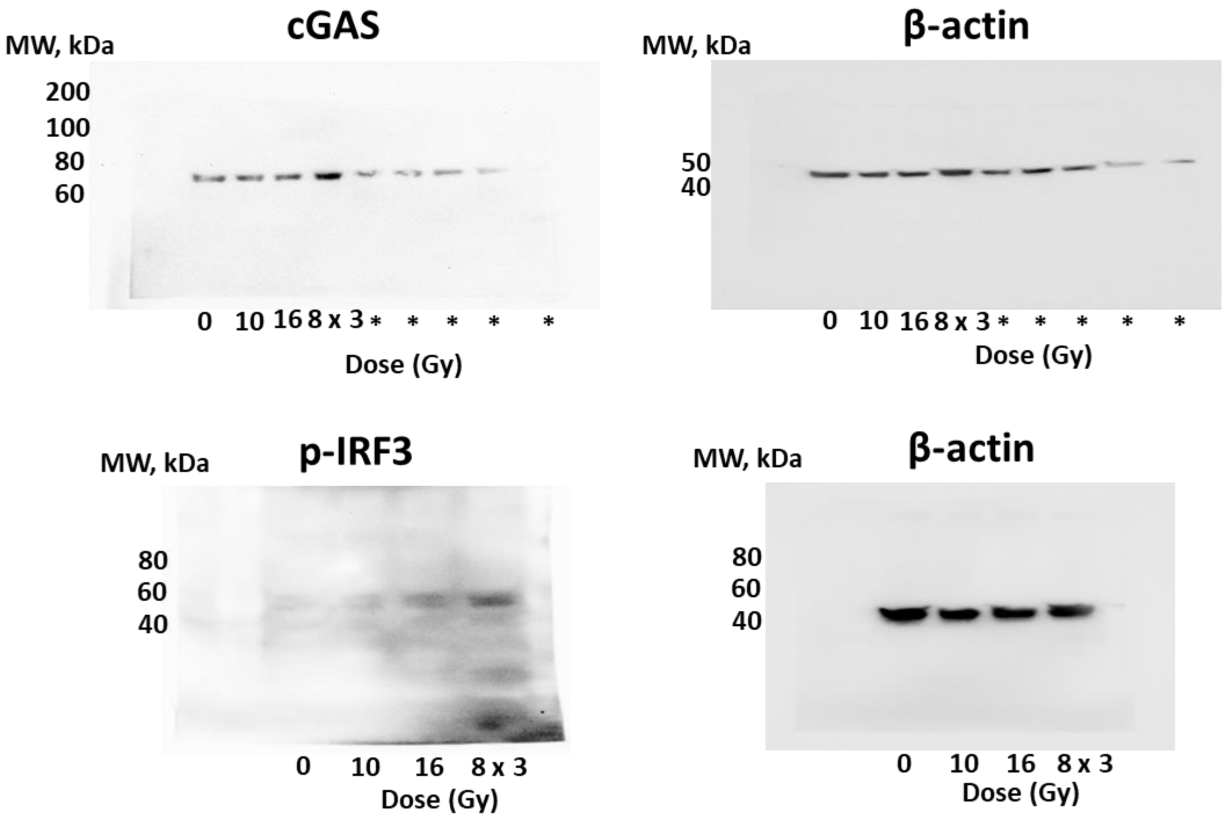

Figure S1. Untrimmed data of western blotting in Figure 3 (b). *: Unrelated samples to this study. Abbreviations; 8 x 3: Irradiation at 8 Gy by three fractions.

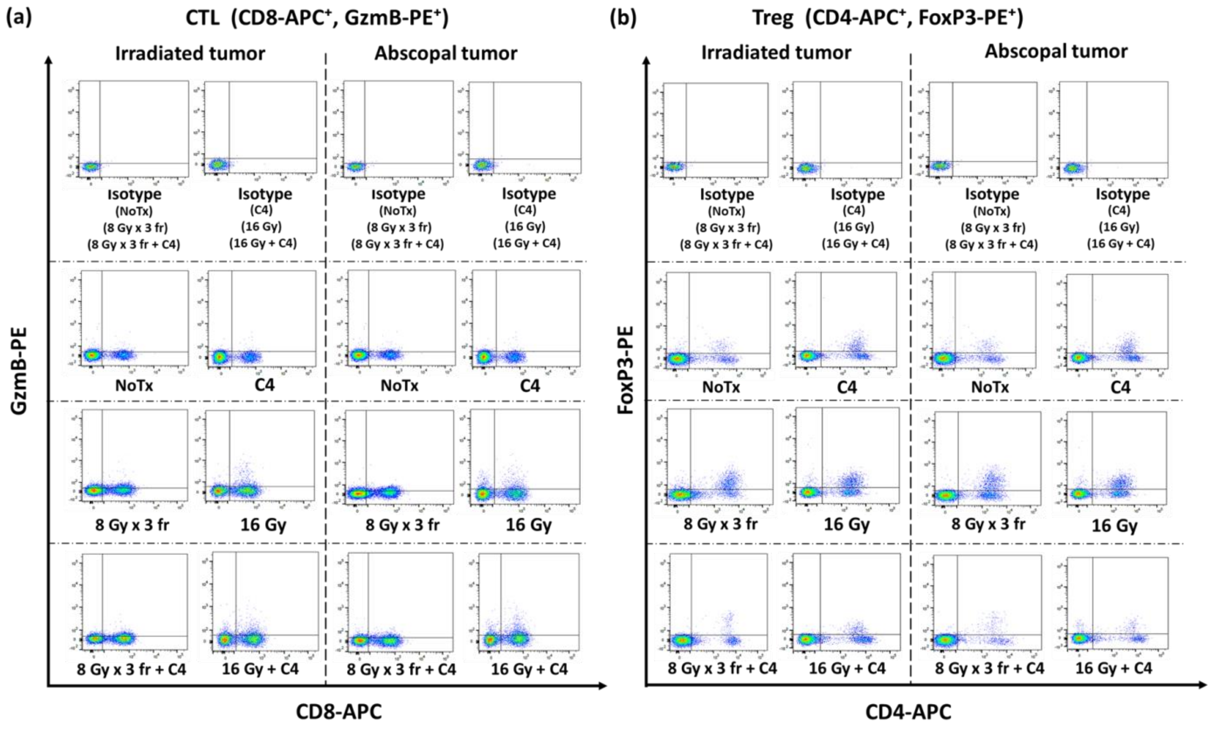

Figure S2. Representative dot plots in flow cytometry analyzing (a) cytotoxic T cells (CD45+,CD8+, and GzmB+), and (b) regulatory T cells (CD45+, CD4+, and FoxP3+).

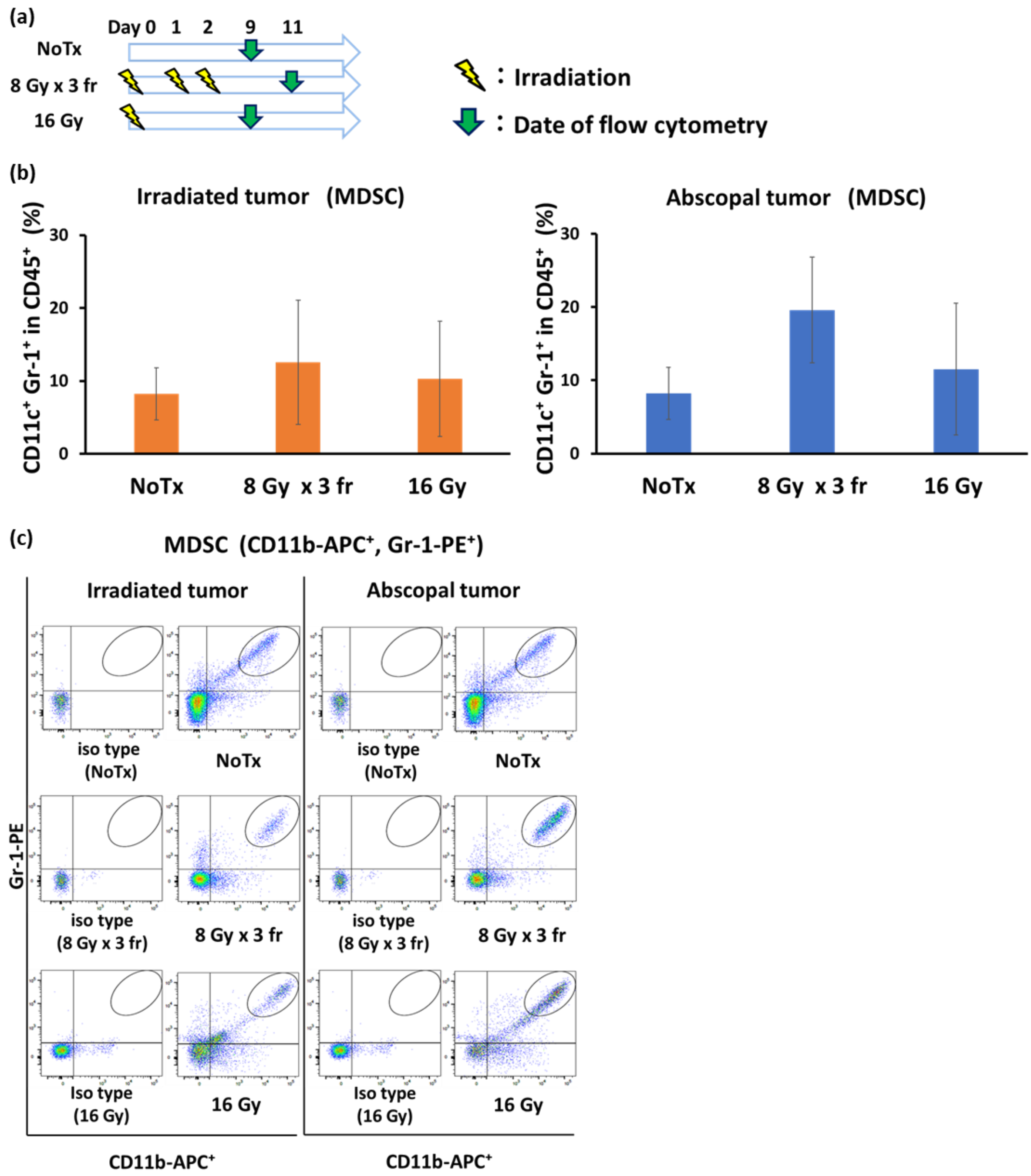

Figure S3. The proportion of MDSC in each group. (a) Schedule of the experiment. Treatments were initiated when the longest diameter of the tumor exceeded 8 mm (Day 0). (b) The proportion of MDSC in each group in irradiated and abscopal tumors. Error bars represent SE. Tukey-kramer HSD test was performed to compare the differences in the proportion of MDSC betweem NoTx, 8 Gy x 3 fr, and 16 Gy). (c) Representative dot plots of MDSC analysis. MDSC is defined as CD11b^{high} Gr-1^{+} cells. Abbreviations; C4: anti-CTLA-4 antibody, MDSC: Myeloid derived suppressor cells, fr: fraction.
