# Supplementary material for: High Dose Local Photon Irradiation Is Crucial in Anti-CTLA-4 Antibody Therapy to Enhance the Abscopal Response in a Murine Pancreatic Carcinoma Model
Source: Cancers (Basel). 2022 Apr 22;14(9):2087. doi: 10.3390/cancers14092087 (PMC9101709; doi:10.3390/cancers14092087)
Supplement: Supplementary file 1 [file cancers-14-02087-s001.zip › cancers-1660363-supplementary Methods S1-done.pdf]

cancers

MDPI

Supplemental Methods S1.

1. Derivation of the biological equivalent dose of 16 Gy in a single fraction

The concept of biologically effective dose (BED) with the LQ model is a well-established method in the field of radiation oncology and biology (Hall EJ. Radiobiology for the Radiologist, 4th ed.; J.B. Lippincott Company: Philadelphia, USA, 1993; pp. 211-229).

The biological equivalent dose can be calculated as follows:

$$BED = nd\left[1 + \frac{d}{\alpha/\beta}\right]$$

where n and d represent the number of fractions and the dose per fraction, respectively. In cancer cells, α/β is assumed as 10. The BED of 16 Gy in a single fraction is then calculated as follows:

$$BED = nd\left[1 + \frac{d}{\alpha/\beta}\right] = 1 \cdot 16\left[1 + \frac{16}{10}\right] = 41.6\,Gy$$

If we deliver a certain dose in 3 fractions that is equivalent to 16 Gy in a single fraction, the following formula is obtained:

$$BED = 3d\left[1 + \frac{d}{10}\right] = 1 \cdot 16\left[1 + \frac{16}{10}\right] = 41.6\,Gy$$

Then d = 8 is obtained.

This provides that 16 Gy in a single fraction is biologically equal to 8 Gy by 3 fractions.
